# Neuroradiology

**DOI:** 10.4103/0971-3026.38512

**Published:** 2008-02

**Authors:** Dhananjay Ghongade, Srikant Moorthy, K.P. Sreekumar, N. K. Prabhu

A 5-year-old boy with complaints of impaired vision, gait disturbance, and delayed milestones was referred for MRI of the brain. On examination, the child was mentally retarded. He had no perception of light. He had pendular nystagmus and convergent squint. He could stand with support. Fundoscopic examination of the eye showed bilateral hyperemic discs with blurred margins and whitish stippling of the retina, with attenuated vessels. There was no evidence of optic atrophy. MRI of the brain was performed [Figures 1 and 2].

**Figure 1:**
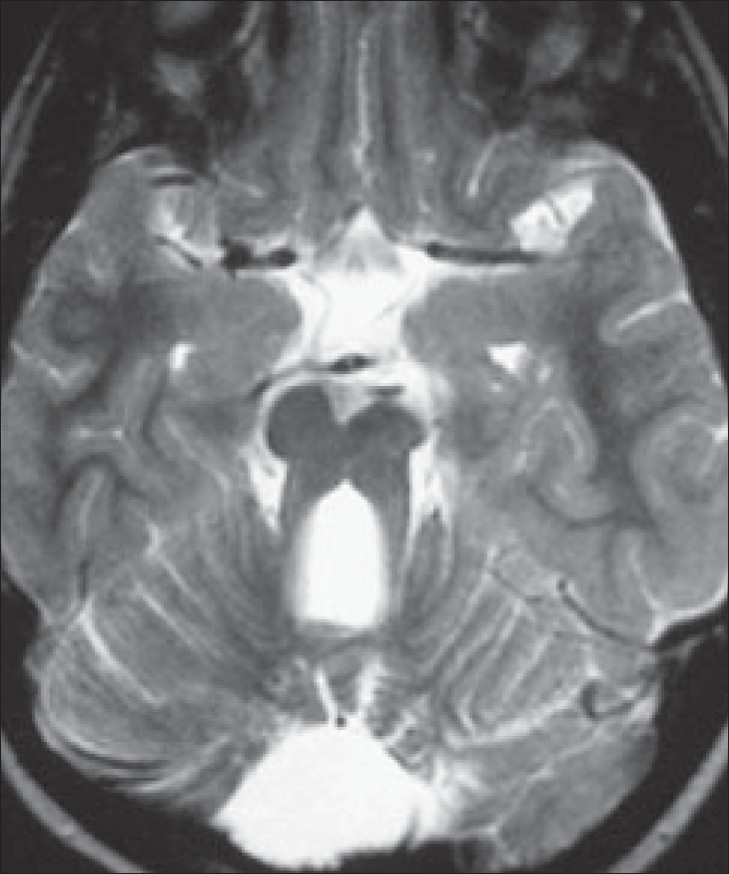
Axial T2W MRI of the brain

**Figure 2:**
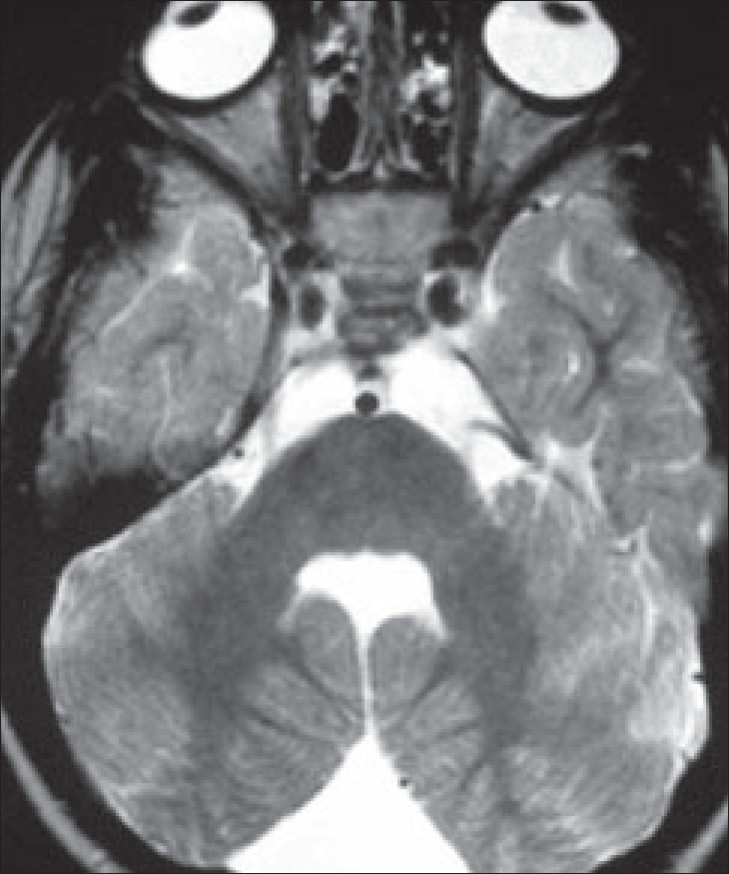
Axial T2W MRI of the brain

## What is the Diagnosis?

### Diagnosis: Joubert's Syndrome: Imaging Findings

**Figure 3:**
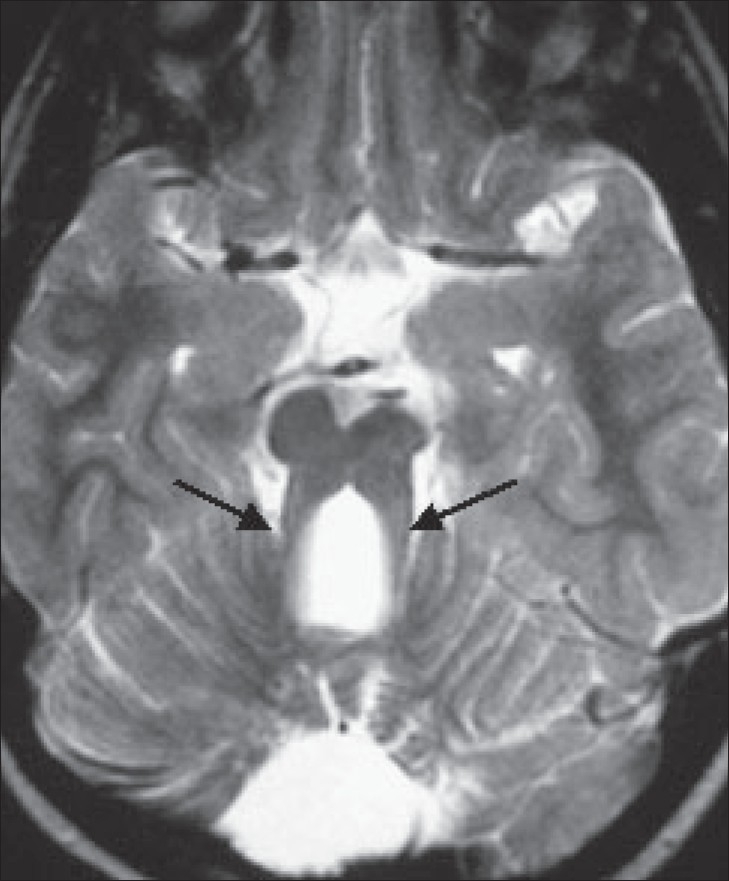
Axial T2W MRI of the brain shows the typical ‘molar-tooth’ configuration of the superior cerebellar peduncles, with a hypoplastic cerebellar vermis

**Figure 4:**
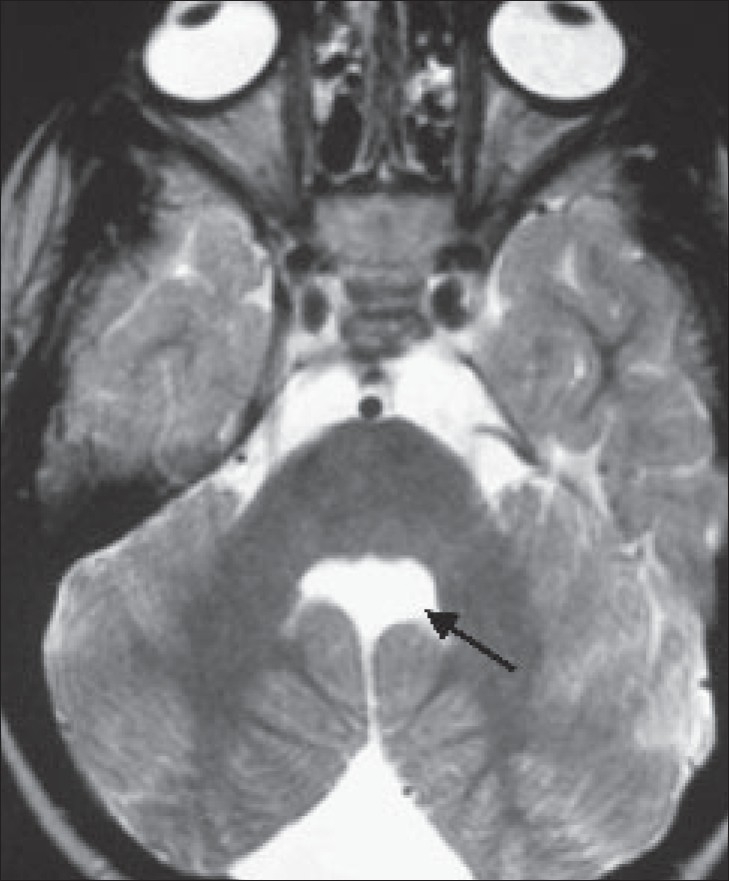
Axial T2W MRI of the brain shows the typical ‘bat-wing’ shaped fourth ventricle. The fourth ventricle is in direct contact with the extracerebellar subarachnoid space and the cerebellar hemispheres are in contact with each other

The MRI of the brain shows small, hypoplastic superior cerebellar peduncles, producing a ‘molar-tooth’ configuration [Figure 3]. The vermis is severely hypoplastic. On axial images, the fourth ventricle shows a ‘bat-wing’ shape [Figure 4]. The fourth ventricle is in direct contact with the extracerebellar subarachnoid space and the cerebellar hemispheres are in contact with each other [Figure 4]. There is no evidence of a posterior fossa cyst or any supratentorial abnormality. These imaging findings, along with the clinical history of delayed milestones and failure to develop vision, are suggestive of Joubert's syndrome.

## Discussion

Joubert's syndrome is an autosomal recessive disorder named after the French neurologist Marie Joubert, who reported five children with mental retardation, episodes of abnormal deep breathing, abnormal eye movements, and ataxia, associated with cerebellar vermian agenesis.[1] These occur due to the absence of decussation of the superior cerebellar peduncles, central pontine tracts, and corticospinal tracts.[1] Dysplasia of the vermis, cerebellar nuclei, and brain stem is probably an associated secondary pathology.

The clinical features include abnormal eye movements - characterized by nystagmus and the inability to smoothly pursue moving objects - with episodes of apnea and hyperpnea and delayed generalized motor development.[2] This syndrome may be associated with other conditions, including retinal coloboma or dystrophy (50%), tongue protrusion (30%), polydactyly (15%), and corpus callosum agenesis. Of those patients with retinal dystrophy, 30% tend to have multicystic renal disease and a poor prognosis.[2]

Typical imaging findings include partial or complete absence of the vermis, hypoplastic cerebellar peduncles, and fourth ventricular deformity. The cerebrum and cerebellar hemispheres are usually unaffected. Other occasional findings include lateral ventricular enlargement due to atrophy, in 6-20% of cases and corpus callosum agenesis in 6-10% cases.[2] The ‘molar-tooth’ sign is due to hypoplasia of the cerebellar peduncles. Severe hypoplasia of the vermis leads to a ‘bat-wing’ appearance of the fourth ventricle. These two signs together are highly suggestive of Joubert's syndrome.

Partial or complete agenesis of the vermis is also present in the Dandy-Walker and Down's syndromes. The Dandy-Walker syndrome is characterized by a posterior fossa cyst, with enlargement of the posterior fossa;[2] hypoplasia of the superior cerebellar peduncles is absent.[3] It is important to differentiate between Joubert's syndrome and Dandy-Walker variants, since genetic counselling is required in Joubert's syndrome. Down's syndrome (trisomy 21) is diagnosed clinically and on karyotyping.[2]

Once a diagnosis of Joubert's syndrome is made, the mother should be closely monitored during subsequent pregnancies for the development of similar features in the fetus, using antenatal USG. Once the diagnosis is made, regular ocular examination for retinal dysplasia and USG for cystic renal lesions should be performed. Due to extreme sensitivity to the respiratory depressant effects of anesthetic agents like nitrous oxide and opioids, these agents should be used with caution during surgery and patients should be monitored closely in the perioperative period.[2]
